# Effect of home-based, overground robotic-assisted gait training on vascular health in people with chronic stroke

**DOI:** 10.3389/fneur.2023.1093008

**Published:** 2023-03-10

**Authors:** James Faulkner, Amy Wright, Keeron Stone, Simon Fryer, Louis Martinelli, Danielle Lambrick, Eloise Paine, Lee Stoner

**Affiliations:** ^1^Department of Sport, Exercise and Health, University of Winchester, Winchester, United Kingdom; ^2^School of Sport, Health and Exercise Science, University of Portsmouth, Portsmouth, United Kingdom; ^3^School of Sport and Health Sciences, Cardiff Metropolitan University, Cardiff, United Kingdom; ^4^School of Sport and Exercise, University of Gloucestershire, Gloucester, United Kingdom; ^5^Hobbs Rehabilitation, Winchester, United Kingdom; ^6^Faculty of Health Sciences, University of Southampton, Southampton, United Kingdom; ^7^School of Sport and Exercise, University of North Carolina, Chapel Hill, NC, United States

**Keywords:** rehabilitation, physical activity, exercise, pulse wave velocity (PWV), robotics, pulse wave analysis (PWA)

## Abstract

Overground robotic-assisted gait training (O-RAGT) has been shown to improve clinical functional outcomes in people living with stroke. The purpose of this study was to identify whether a home-based O-RAGT program, in combination with usual care physiotherapy, would demonstrate improvements in vascular health in individuals with chronic stroke, and, whether any changes in vascular outcomes would be sustained 3 months after completing the program. Thirty-four participants with chronic stroke (between 3 months and 5 years post-stroke) were randomized to either a 10-week O-RAGT program in combination with usual care physiotherapy, or to a usual care physiotherapy only control group. Participants' (*n* = 31) pulse wave analysis (PWA), and regional [carotid-femoral pulse wave analysis (cfPWV)] and local (carotid) measures of arterial stiffness were assessed at baseline, post-intervention, and 3-month post-intervention. Analysis of covariance demonstrated a significant reduction (improvement) in cfPWV between BL and PI for O-RAGT (8.81 ± 2.51 vs. 7.92 ± 2.17 m/s, respectively), whilst the control group remained unchanged (9.87 ± 2.46 vs. 9.84 ± 1.76 m/s, respectively; *p* < 0.05; ηp^2^ = 0.14). The improvement in cfPWV was maintained 3 months after completing the O-RAGT program. There were no significant Condition by Time interactions for all PWA and carotid arterial stiffness measures (*p* > 0.05). A significant increase in physical activity, as determined by the time spent stepping, was observed for O-RAGT between baseline and post-intervention assessments (3.2 ± 3.0–5.2 ± 3.3%, respectively) but not for CON (*p* < 0.05). The improvement in cfPWV, in combination with an increase in physical activity whilst wearing the O-RAGT and concomitant reduction in sedentary behavior, are important positive findings when considering the application of this technology for “at home” rehabilitation therapy for stroke survivors. Further research is needed to determine whether implementing “at home” O-RAGT programs should be a part of the stroke treatment pathway.

**Clinical trial registration:**

https://clinicaltrials.gov, identifier NCT03104127.

## Introduction

Globally, stroke is the second leading cause of mortality and lost disability adjusted life years (1). Stroke recurrence and mortality are impacted by several modifiable risk factors, and as such are amenable to secondary prevention strategies (2). Physical activity (PA) and exercise and are efficacious modifiable risk factors that are widely encouraged in stroke survivors as they have been shown to improve physical fitness (oxygen uptake; 95% confidence interval (CI) 2.98–3.83 mL·kg·min^−1^ higher), enhance aspects of physical function (3 m timed-up-and-go test; 95%CI 2.05 to 4.78 s faster) (3, 4), as well as reduce recurrent stroke (5) and cardiovascular disease risk (6). Recovering the ability to walk following a stroke is also a priority in this population (3). For people living with stroke who have functional limitations, robotic-assisted gait training has been shown to improve walking capacity, walking speed and motor performance (7).

Over-ground robotic-assisted gait training devices (O-RAGT) allow the patient to walk in a real-world environment, enabling substantial kinematic variability while ensuring successful task execution (8). The home-based use of O-RAGT may contribute to the formation of habits that lead to long-term behavior change as people are able to use such devices in a familiar context (9). Previous research from our laboratory found clinicially meaningful improvements in functional outcomes (i.e., 6-min walk test, balance) after a 10-week daily, home-based, rehabilitation program using O-RAGT, in the form of a wearable robotic knee orthosis in chronic stroke patients (10). Furthermore, there was an increase in PA (steps taken) on completion of the O-RAGT which was maintained for a further 3 months after completion of the O-RAGT program. Whilst it is known that O-RAGT led to sustained improvements in PA and physical function (10), it is unknown whether it also leads to sustained improvements in markers of cardiovascular health, including blood pressure and arterial stiffness (9). This is important considering that arterial stiffness is a strong independent risk factor for cardiovascular disease (11).

Elevated brachial blood pressure, an important risk factor for stroke (12), is widely cited as a marker that needs to be controlled post-stroke by pharmacological and lifestyle management (13), which could include the engagement in exercise interventions (14). However, central haemodynamic components such as aortic arterial stiffness are better predictors of vascular disease than brachial blood pressure (15). This is because measures of arterial stiffness, such as pule wave velocity (PWV), integrate the damage of risk factors on the arterial wall over a long period, whereas traditional risk factors, including blood pressure, hyperglycaemia and dyslipidaemia, can acutely fluctuate (16). As the aortic walls stiffen, PWV increases which causes a rise in central systolic pressure and a widening of aortic pulse pressure (17). In ischemic stroke, low aortic stiffness, as measured by carotid-femoral PWV (cfPWV) is associated with early favorable outcome, independently of other known prognostic factors (17). However, whether a walking-based O-RAGT program elicits favorable changes in aortic arterial stiffness in people with chronic stroke is unknown.

The purpose of this study was to identify whether: (i) a home-based O-RAGT program, in combination with usual care physiotherapy, would demonstrate improvements in cardiovascular health (e.g., cfPWV, blood pressure) in individuals with chronic stroke, and, (ii) any changes in cardiovascular health outcomes would be sustained for 3 months. It was hypothesized that regular participation in a 10-week O-RAGT program would improve vascular health in individuals living with stroke.

## Materials and methods

This study was a parallel group, randomized controlled clinical trial, reported in accordance with Consolidated Standards of Reporting Trials (CONSORT) guidelines (18). The study protocol received institutional human research ethics approval and was registered with ClinicalTrials.gov Protocol Registration and Results System (NCT03104127; https://clinicaltrials.gov/ct2/show/NCT03104127).

### Participants

Participants with chronic stroke (>3 months since stroke diagnosis) were recruited from a single neuro-physiotherapy practice (Hobbs Rehabilitation, Winchester, UK). All participants were diagnosed with stroke by a specialist neurologist/stroke consultant from a UK National Health Service Trust and had completed rehabilitation activities (i.e., inpatient and outpatient) in accordance with recommended guidelines (19). Written informed consent was obtained from all participants prior to the commencement of the study.

Inclusion criteria included: Individuals between 3 months and 5 years post-stroke, who were living in the community, medically stable, and cognitively capable, able to stand and step with an aid or with assistance (defined as a Functional Ambulation Categories between 2 and 5) (20), and who were receiving physiotherapy or attending a community-based, stroke support group at the time of study enrolment. Exclusion criteria included: body mass index (BMI) ≥40 kg/m^2^, major arrhythmias, unresolved deep vein thrombosis, recent fractures of the symptomatic limb, open wounds, severe osteoporosis, and/or individuals who were non-weight bearing.

### Experimental design

Participants were tested between 07:00 and 10:00 am in the physiology laboratory at the University of Winchester. Participants refrained from intense physical activity for 24 h prior to testing, and could only consume water for the 12 h before testing. Following an initial Functional Ambulation Classification and Modified Rankin Scale assessment to provide an indication of the degree of disability, participants lay supine for 15 min. Thereafter, pulse wave analysis (PWA), and regional (cfPWV) and local (common carotid) measures of arterial stiffness were assessed. Participants were randomized using covariate adaptive randomization (21) to either a 10-week home-based O-RAGT program, which included weekly “usual care” physiotherapy, or to a 10-week “usual care” physiotherapy only program (CON). Randomization involved sequentially assigning participants to O-RAGT or control by taking into account their age (age?70 vs. <70 years), systolic blood pressure (SBP ≥ 160 vs. < 160 mmHg) and time since stroke (< 12 vs. ≥ 12 months). Identical assessments were completed at baseline, post intervention (PI) and 3-months post-intervention (3PI). Participants and researchers collecting outcome data were aware of the allocated treatment condition, however, data analysts were blinded to the allocation.

### Outcome measures

#### Pulse wave velocity

The SphygmoCor XCEL device enables simultaneous assessment of proximal and distal arterial waveforms using a tonometer and volume-displacement cuff, respectively, to determine arterial pulse transit time. Carotid–femoral pulse transit time was measured as the time between diastolic feet of the proximal (tonometer) and distal (cuff) arterial pulse waveforms (22). PWV was calculated by dividing pulse transit time by arterial path length, or PWV distance. For cfPWV, the tonometer was placed on the left carotid artery and the oscillometric cuff on the left thigh at the level of the femoral artery. The carotid–femoral was estimated by measuring the linear distance from the suprasternal notch to the top of the cuff at the center line of the leg and subtracting the distance from the suprasternal notch to the carotid artery. Accordingly, cfPWV was calculated as: cfPWV = carotid-femoral distance/carotid-femoral pulse transit time. Two measurements were taken, but if a difference of > 0.5 m·s was recorded, a third measure was completed and an average taken of the closest two.

#### Pulse wave analysis

For PWA, oscillometric pressure waveforms were recorded on the left upper arm by a single observer using the SphygmoCor XCEL device (AtCor Medical, Sydney, Austrailia), following standard manufacturer guidelines (23). Each single measurement cycle consisted of a 60 s brachial blood pressure recording followed by a 10 s sub-systolic recording. A corresponding aortic pressure waveform was then generated using a validated transfer function (24), from which central systolic blood pressure (cSBP), augmentation index (AIx) and augmentation pressure, were derived. Peripheral blood pressures and mean arterial pressure were also measured. Two measurements were taken, but if a difference of > 5 mmHg in peripheral blood pressure and a difference of > 4% for AIx was recorded (as per manufacturer guidelines), a third measure was completed and an average taken of the closest two. Measurements were taken at heart level to ensure no changes in AIx were found due to alterations in arm angle. Augmentation index was normalized to a heart rate of 75 bpm (AIx75).

#### Common carotid arterial stiffness

A trained ultrasound operator with extensive experience (>10 years) collected all common carotid arterial stiffness measurements using a portable uSmart 3,300 Ultrasound system (Terason, USA) equipped with a 13–6 MHz bandwidth transducer that provided high resolution brightness mode measurements. The left common carotid artery of the participants was examined, in a supine position, and with their head tilted at 45° (angled to the right) on completion of PWA and PWV measurements. The left common carotid artery was assessed 1–2 cm beneath the bifurcation (25). Magnification and focal zone settings were adjusted to optimize the image of the proximal and distal vessel walls, while ultrasound global (e.g., acoustic output, gain, dynamic range, gamma and rejection) and probe-dependent (e.g., zoom factor, edge enhancement, frame averaging and target frame rate) settings were standardized (26). Three 10 s video recordings, captured at 30 frames·s, were obtained during which participants were asked to hold their breath. Videos were recorded using external video capturing software (LiteCam HD, Englewood Cliffs, NJ, USA). The video clips were analyzed offline using automated edge-detecting software (FMD Studio, Quipu, Italy). Custom written Excel Visual Basic code was used to fit peaks and troughs to the diameter waveforms in order to calculate measures of arterial stiffness, compliance and distensibility.

### Accelerometry

Participants wore an ActivPAL3™ device (PAL Technologies Ltd., Glasgow, Scotland) for seven consecutive days and nights at baseline, PI and 3PI. The ActivPAL3 device was wrapped in a protective Tegaderm™ (3M, St Paul, USA) and attached to the anterior aspect of the upper third of the thigh, on the asymptomatic side. The ActivPAL3 provided a daily measure of the: (1) percentage of time spent sitting or lying, (2) percentage of time spent standing, (3) percentage of time spent stepping, and (4) step counts.

### O-RAGT device

The O-RAGT device (Alter-G, Bionic Leg orthosis, Fremont, CA, USA) is a battery-operated, externally-wearable, dynamic device worn by stroke patients during rehabilitation. The device provides sensory inputs (i.e., auditory and sensory feedback), mobility assistance for users with reduced lower-limb function, and is fitted and worn in a manner similar to an orthopedic knee brace. The orthosis shell functions as the user interface that transfers the assistive torque to the human body, while an actuation unit assists the movement of the limb.

### O-RAGT program

Participants were familiarized with the O-RAGT device before commencing the 10-week home-based program. Participants were encouraged to undertake at least 30 min per day of continuous or non-continuous bouts of walking and sit-to-stand exercises, at a moderate ratings of perceived of exertion (RPE 12–13). There was no maximum daily wear-time. O-RAGT settings associated with a participant's weight, assistance, resistance, threshold and knee extension angle settings were individualized and re-assessed every 2 weeks. Participants reported their number of steps, duration of use, activities undertaken and RPE for each day of activity. During this time, participants also continued their “usual care.”

### Usual care physiotherapy

Participants in both the control group and O-RAGT program undertook one-to-one, “usual care” physiotherapy sessions for the duration of the study. This included stretching and muscle strengthening exercises, functional movement activities (e.g., walking, step-ups, sit-to-stand) and soft-tissue massage. There were also group therapy activities which were based on the same principles but with less therapist engagement. For the duration of the 10-week program participants were advised to engage in at least 30 min of physical activity each day, undertaking similar functional movement patterns as those reported above.

### Data analysis

Demographical and clinical comparisons between Conditions (O-RAGT, CON) was undertaken at baseline with independent sample *t-*tests (e.g., age, time since stroke, Functional Ambulation Classification, Modified Rankin Scale, PWA, cfPWV and carotid arterial stiffness outcomes) and chi-square tests (e.g., sex, stroke diagnosis), as appropriate.

To assess the effect of the O-RAGT intervention on the aforementioned regional and local hemodynamic properties, mixed model, two-factor analysis of covariance (ANCOVA), Condition (O-RAGT, control) x Time (BL, PI, 3PI), adjusted for baseline measures and age, were used to assess all PWA, cfPWV, carotid artrial stiffness and accelerometry outcomes. For PWV analysis, Mean Arterial Pressure was also used as a covariate. Partial eta squared (ηp^2^) was used to demonstrate the strength of the effect of exercise on the various outcome measures with 0.0099, 0.0588 and 0.1379 representing a small, medium and large effect, respectively (27). Alpha was set at 0.05. Statistical analyses were performed using Statistical Package for Social Sciences version 26 (SPSS, Inc., Chicago, IL, USA). All data are reported as means (s.d.), unless otherwise specified.

## Results

**Figure 1:**
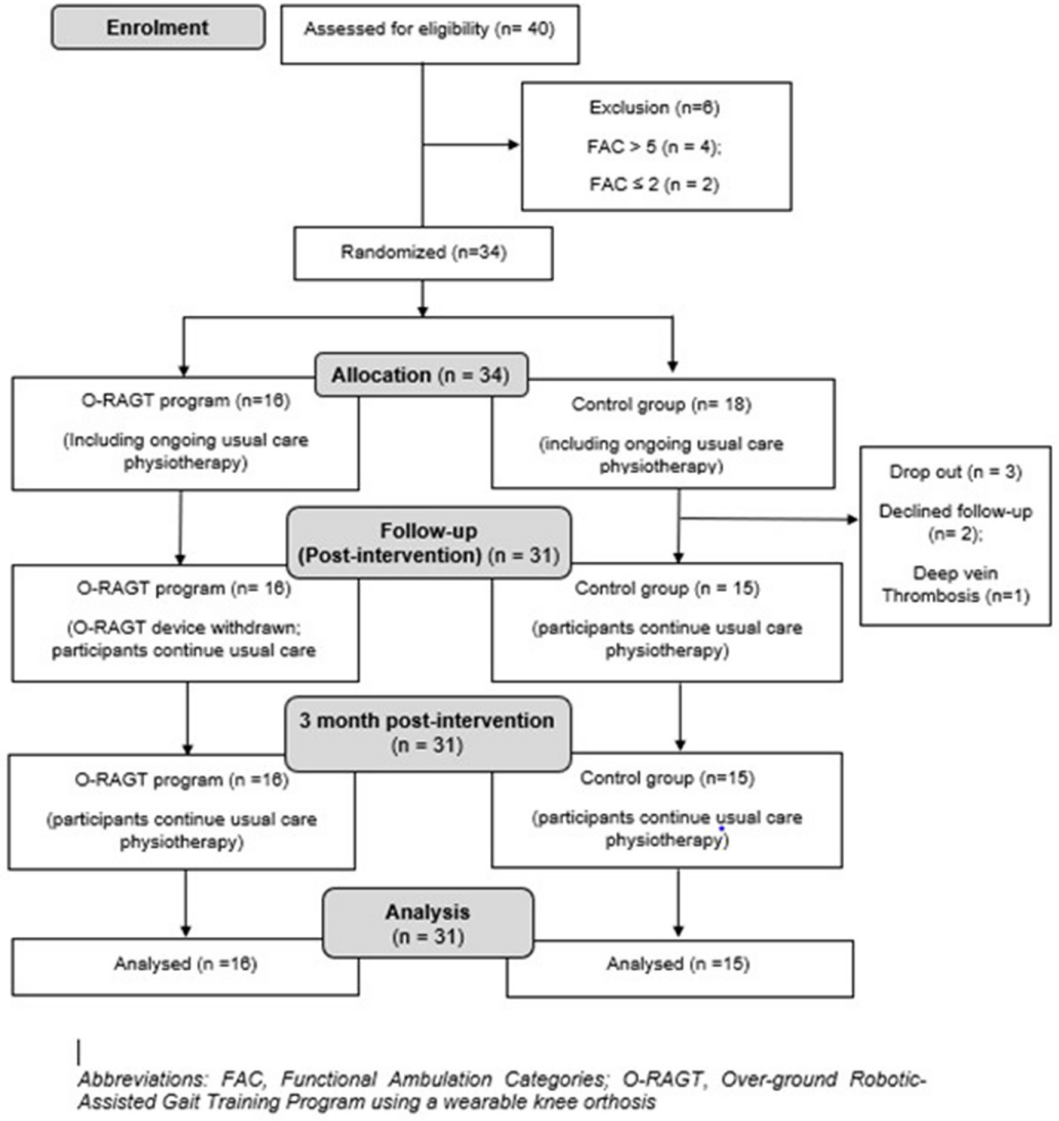
Consort statement.

Participant recruitment and retention are presented in Figure 1. The 31 participants who attended all three assessments (BL, PI, 3PI) were generally older males who had been living with stroke for between 1 and 5 years (Table 1). For O-RAGT, there was an increase in daily wear time (50 ± 20–72 ± 41 mins) and steps taken with the robotic device (887 ± 520–945 ± 542 steps), and decreases in RPE (12.8 ± 2.2–10.4 ± 3.2), from the first to the last week of the O-RAGT intervention, respectively. There were no adverse events whilst participants wore the O-RAGT device.

**Table 1:** Participant demographics at baseline.

**Demographic**		**O-RAGT**		**CON**		***p***
		***n***	**%**	***n***	**%**	
Sex	Male	14	88	13	87	0.945
	Female	2	12	2	13	
Age (years)		59.6 ± 10.1		64.2 ± 10.7		0.228
Stroke diagnosis	Ischemic	15	94	13	87	0.505
	Hemorrhagic	1	6	2	13	
Hemiparetic side	Left	11	69	9	60	0.611
	Right	5	31	6	40	
Orthotic^*^	Yes	9	56	10	66	0.955
	No	7	44	5	34	
Walking aid^**^	Yes	14	88	12	80	0.570
	No	2	12	3	20	
Time since stroke (months)		31 ± 19		28 ± 21		0.679
FAC		3.4 ± 1.0		3.3 ± 1.1		0.793
MRS		3.3 ± 0.6		3.4 ± 0.7		0.672

Age, time since stroke, FAC, and MRS are presented as mean ± SD. All other demographics are presented as total number and percentage.

CON, Control group; FAC, Functional Ambulation Categories; MRS, Modified Rankin Scale; O-RAGT, Over-ground-Robotic Assisted Gait Training.

* Orthotic refers to a soft or hard foot and/or ankle brace.

** Walking aid refers to use of a walking stick, tripod or quadripod.

There were no differences at BL between Conditions for all outcomes except for cfPWV (Table 2). ANCOVA demonstrated a significant Condition by Time interaction for cfPWV (*p* < 0.05; Partial η^2^ = 0.224; Table 2). The O-RAGT group demonstrated a significant reduction (improvement) in cfPWV between BL and PI, whilst the CON was unchanged. The improvement in cfPWV was maintained at 3PI for O-RAGT. There were no significant Condition by Time interactions for all other PWA or arterial stiffness outcomes (*p* > 0.05; Table 2, Supplementary Table A).

**Table 2:** PWA, cfPWV and local arterial stiffness outcome measures reported at baseline (BL) and post-intervention (PI, 3PI) for O-RAGT and control (CON) conditions.

		**Assessment**			***Condition x Time interaction***		
		**BL**	**PI**	**3PI**	**F**	***p***	η**p**^2^
SBP (mmHg)	O-RAGT	139 ± 14	133 ± 14	135 ± 13	2.161	0.123	0.061
	CON	142 ± 17	143 ± 21	142 ± 19			
DBP (mmHg)	O-RAGT	83 ± 12	81 ± 11	82 ± 10	0.086	0.917	0.003
	CON	81 ± 9	80 ± 8	81 ± 10			
cSBP (mmHg)	O-RAGT	130 ± 13	125 ± 13	127 ± 12	1.207	0.306	0.040
	CON	130 ± 16	130 ± 18	128 ± 18			
cDBP (mmHg)	O-RAGT	84 ± 11	82 ± 11	82 ± 10	0.356	0.701	0.012
	CON	84 ± 10	81 ± 10	81 ± 9			
cPP (mmHg)	O-RAGT	46 ± 12	44 ± 10	45 ± 11	1.428	0.248	0.047
	CON	48 ± 14	49 ± 15	48 ± 13			
AIx75	O-RAGT	27.8 ± 13.4	24.6 ± 11.5	26.4 ± 12.7	1.169	0.317	0.034
	CON	26.1 ± 8.5	25.1 ± 8.9	24.2 ± 8.7			
MAP (mmHg)	O-RAGT	101 ± 11	97 ± 12	97 ± 10	0.188	0.829	0.005
	CON	99 ± 10	98 ± 11	98 ± 11			
cfPWV (m/s)	O-RAGT	8.81 ± 2.51	7.92 ± 2.17	7.89 ± 2.30	4.261	0.023^*^	0.135
	CON	9.87 ± 2.46	9.84 ± 1.76	9.87 ± 1.77			
β-stiffness	O-RAGT	10.5 ± 4.9	8.7 ± 3.6	9.3 ± 3.7	1.147	0.325	0.038
	CON	9.0 ± 2.2	9.4 ± 2.2	9.3 ± 3.0			

AIx75, AIx at 75 b·min^−1^; BL, Baseline; cfPWV, Carotid-femoral pulse wave velocity; cDBP, Central diastolic blood pressure; CON, Control; cPP, Central pulse pressure; cSBP, Central systolic blood pressure; DBP, Diastolic blood pressure; MAP, Mean arterial pressure; O-RAGT, Over-ground robotic-assisted gait training; PI, Post-intervention; SBP, Systolic blood pressure; 3PI, three-month post-intervention.

* Significant Test x Condition interaction (p < 0.05).

For the accelerometry outcomes, a significant Condition by Time interaction was observed for the time spent stepping (*p* < 0.05; Supplementary Table B). The O-RAGT group demonstrated a significant increase in time spent stepping between BL and PI. There were no significant Condition by Time interactions for all other accelerometry outcomes (*p* > 0.05; Supplementary Table B).

## Discussion

This study demonstrated improvements in cfPWV in chronic stroke survivors following a combination of daily, home-based O-RAGT, in the form of a wearable robotic knee orthosis, and usual care physiotherapy. The improvement in cfPWV which was observed on completion of the 10 week program was maintained three-months post-intervention (3PI). The improvement in cfPWV, in combination with an increase in wear-time and physical activity whilst wearing the O-RAGT, are important positive findings when considering the application of this technology for “at home” rehabilitation therapy for stroke survivors.

### Short-term effect of O-RAGT (baseline to post-intervention)

Carotid-femoral PWV predicts mortality in patients with essential hypertension (28) and is a strong predictor of cardiovascular disease in a range of clinical populations (29). Past research has shown significant improvements in cfPWV following 12 weeks of supervised aerobic or resistance training in patient populations when compared to usual care (30). Our study demonstrated that cfPWV decreased by, on average, 0.91 m/s (~12%) in the O-RAGT group at PI, compared to a 0.12 m/s (~1%) in the control group. This is highly encouraging as a 1 m/s reduction in cfPWV is the minimal clinically important difference, and is strongly associated with decreased cardiovascular disease risk (29). Although O-RAGT did not quite elicit this minimal clinically important difference, the statistically significant interaction and the large effect size (ηp^2^ = 0.135) indicates a robust and promising impact on regional arterial stiffness.

In the present study there were no statistical changes in central haemodynamic (PWA) or local carotid arterial stiffness parameters (Table 2; Supplementary Table A). Past research has demonstrated that aerobic training interventions typically elicit reductions in SBP (95% CI) of up to 5.0 mmHg (31), while during a large-scale analysis of randomized trials, a 5 mmHg reduction of SBP following pharmacological treatment reduced the risk of major cardiovascular events by ~10% (32). Accordingly, although not statistically significant, the ~6 mmHg reduction in SBP and cSBP for O-RAGT participants is comparable with prior literature and highly encouraging given the limited mobility of the population and the low-intensity O-RAGT intervention implemented. It is notable that unlike cfPWV, local carotid artery stiffness did not change in response to O-RAGT, but this is perhaps not surprising given that regional measures of arterial stiffness summate a larger portion of the arterial tree (e.g., cfPWV) and thefore may better detect the impact of cardiovascular disease risk factors (i.e., blood pressure and physical activity). Further, it is well-recognized that regional and local measures of arterial stiffness are not always closely associated (12). Although not significant, the average changes in local carotid artery stiffness, compliance and distensibility for O-RAGT particpants between BL and PI were −17, 10, and 16%, respectively. Past research has shown larger changes in common carotid arterial compliance (17%) and distensibility (22%) in people with stroke who engaged in a moderate to high intensity exercise program (33). Woolley et al. (33) also observed reductions in SBP and DBP of 6 and 12%, respectively, and stated that as these changes were concomitant with the reduction in carotid artery stiffness, it may be suggested that reduced blood pressures had greater influence on local (carotid) arterial stiffness than potential modifications to the elastic properties of the vessel.

### Longer-term effect of O-RAGT (post-intervention to 3-month post-intervention)

Long-term outcomes are of utmost importance when evaluating the clinical importance of interventions. An important characteristic of successful behavior change is that individuals continue to engage in lifestyle modifications once the stimulus (i.e., use of the O-RAGT device) has been removed. A recent meta-analysis for stroke patients revealed that end-of-intervention benefits gained from regular physical fitness training do not persist following completion of an intervention (4). In non-stroke populations, some exercise studies have shown that following 1-month cessation of an exercise intervention, PWV values revert back to pre-intervention baseline levels (34, 35). However, in our study the improvement in cfPWV at PI was maintained at 3PI. This finding may be underpinned by the fact that the increase in physical activity (e.g., time spent stepping) observed between BL and PI was sustained between PI and 3PI (Supplementary Table B). For example, participants undertook an additional ~1,700 steps per day at the time of the PI assessment compared to BL (~39% improvement), which was generally maintained at the 3PI assessment. This positive change in habitual activity patterns may have important practical implications for the adoption of over-ground, lower-limb robotic technology in the rehabilitation of stroke patients. As we recruited a chronic stroke population, it will be of interest to see whether similar changes in cfPWV and habitual activity patterns occur when implementing O-RAGT interventions with acute stroke patients (≤ 3 months), and whether such devices are beneficial for individuals who do not receive ongoing rehabilitation.

The encouraging findings surrounding cfPWV is unique as the O-RAGT program focused on walking, a low-intensity activity, with RPEs of 11 to 13 typically recorded in the activity diaries (Supplementary Table B). Past research has often shown favorable changes in PWV when training interventions have prescribed moderate to vigorous volumes of physical activity (36, 37). However, low-intensity exercise may be more achievable and sustainable than higher intensity programs as feelings of enjoyment and wellbeing are strong motives for continued participation (38). Ekkekakis and colleagues' review into the pleasure and displeasure people feel whilst exercising reported that pleasure is reduced mainly above the ventilatory or lactate threshold, but that pleasant percpetions are often observed below such threshold intensities (39), which would likely have been the case in our study. Due to the encouraging findings of the present study and those associated with functional outcome measures (10), measures of enjoyment during and following robotic technology use at low-intensities of physical activity should be monitored in both the short- and longer-term (e.g., 12 months PI), as this type of technology and O-RAGT program could have a substantial impact in aiding the recovery of chronic stroke surivors.

### Strengths and limitations

In order to contextualize the present findings, specific limitations must be addressed. Firstly, the small sample size was determined based on a primary outcome measure which was not a focus in this study (6-min walk test) (10). However, an a priori sample size calculation based on the cfPWV reported between groups at PI demonstrated that a sufficienct sample size was recruited (*n* = 13 per group). Secondly, regional (cfPWV) and local (carotid) measures of arterial stiffness were only investigated on participants' left-side. As the stroke diagnosis (and hemisphere affected) varied between participants (Table 1), the assessment of regional and local stiffness measures on both the right and left-side may have been informative, particularly for those participants for whom the right carotid artery may have been symptomatic. Thirdly, participants were recruited from an independent neuro-physiotherapy practice which could be a determining factor to whether a home-based program is successful. The selected population were likely to be highly motivated to engage in rehabilitation due to the costs associated with engaging in physiotherapy with an independent provider. The total dosage of physical activity in the O-RAGT condition was likely higher than the control condition and could have also been a reason for the observed findings. Finally, findings should be interpreted with caution as multiple analyses inflate the risk of type I error, while researchers responsible for collecting outcome data were not blinded to group allocation. Strengths to the study included the use of gold-standard non-invasice measures of arterial stiffness, the inclusion of a 3-month PI assessment, and the implementation of a home-based exercise program which may have enabled participants to undertake a higher volume of walking as the participants could wear the O-RAGT device at any time or day during the program period. The observed increases in habitual physical activity could help prevent secondary complications associated with cardiovascular disease and future cardio- or cerebro-vascular events (i.e., reducing strokes) if such programs are implemented over the longer-term.

In conclusion, the present study has demonstrated that participation in a 10-week, home-based, O-RAGT program, in combination with weekly, usual care physiotherapy, can elicit greater improvements in regional (cfPWV) measures of arterial stiffness in people with stroke than “usual care” alone. Importantly, the changes reported in cfPWV were maintained at 3PI assessment suggesting this may be a sustainable and efficacious treatment option once access to the O-RAGT device has been removed. Individuals randomized to the O-RAGT program also demonstrated increases in physical activity which could have the potential to improve quality of life. However, larger randomized controlled trials are required to identify whether the use of O-RAGT is appropriate to recommend as a part of usual care, while further research is also needed to determine whether implementing “at home” O-RAGT programs should be a part of the stroke treatment pathway.

## Data availability statement

The raw data supporting the conclusions of this article will be made available by the authors, without undue reservation.

## Ethics statement

The studies involving human participants were reviewed and approved by University of Winchester (RKE/10/2015-16). The patients/participants provided their written informed consent to participate in this study.

## Author contributions

JF and LM conceptualized the study. JF, AW, KS, SF, DL, and EP collected the data for the study. JF and LS were responsible for the formal analysis. JF was responsible for the original draft preparation. All authors reviewed, edited, approved the final manuscript, read, and agreed to the published version of the manuscript.
