# Supplementary material for: Effect of home-based, overground robotic-assisted gait training on vascular health in people with chronic stroke
Source: Front Neurol. 2023 Mar 10;14:1093008. doi: 10.3389/fneur.2023.1093008 (PMC10036898; doi:10.3389/fneur.2023.1093008)
Supplement: Supplementary file 1 [file Table_1.pdf]

Supplementary Table A: Additional PWA, regional and local arterial stiffness outcome measures reported at baseline (BL) and post-intervention (PI, 3PI) for O-RAGT and control (CON) conditions

		Assessment			*Condition x Time interaction*		
		**BL**	**PI**	**3PI**	**F**	***p***	**ηp^2**
**AP**	**O-RAGT**	15.6 ± 7.9	13.6 ± 7.5	15.1 ± 7.1	2.919	0.061	0.081
	**CON**	17.2 ± 8.0	17.1 ± 7.2	16.2 ± 6.9			
**AIx**	**O-RAGT**	33.3 ± 13.3	30.1 ± 12.2	31.6 ± 11.7	2.447	0.094	0.069
	**CON**	33.9 ± 8.6	33.3 ± 8.8	32.0 ± 8.1			
**HR ($b \cdot min^{-1}$)**	**O-RAGT**	63 ± 12	62 ± 11	61 ± 12	1.368	0.262	0.040
	**CON**	61 ± 14	61 ± 14	61 ± 14			
**Δd (mm)**	**O-RAGT**	0.35 ± 0.11	0.39 ± 0.12	0.38 ± 0.12	0.399	0.673	0.014
	**CON**	0.39 ± 0.13	0.41 ± 0.17	0.40 ± 0.18			
**CC ($mm^2 kPa^{-1}$)**	**O-RAGT**	0.98 ± 0.41	1.09 ± 0.42	1.09 ± 0.46	0.550	0.580	0.019
	**CON**	1.04 ± 0.29	1.04 ± 0.29	1.06 ± 0.50			
**DC ($10^{-3} kPa^{-1}$)**	**O-RAGT**	21.8 ± 8.3	25.9 ± 8.7	25.0 ± 9.1	0.877	0.421	0.029
	**CON**	22.8 ± 6.1	22.5 ± 7.3	23.6 ± 8.7			

Abbreviations: AP, Augmentation pressure; Aix, Augmentation index; BL, Baseline; CC, Compliance coefficient; cfPTT, CON, Control; DC, Distensibility coefficient; Δd, Distension; HR, Heart rate; O-RAGT, Over-ground robotic-assisted gait training; PI, Post-intervention; PP3PI, three-month post-intervention

Supplementary Table B: Mean (± SD) accelerometry data for O-RAGT and control at BL, PI and 3PI

		Assessment			*Condition x Time interaction*		
		**BL**	**PI**	**3PI**	**F**	***p***	**ηp^2**
**Time spent seated/supine (%)**	**O-RAGT**	86.3 ± 10.6	83.4 ± 11.2	85.2 ± 9.6	0.980	0.337	0.035
	**CON**	84.6 ± 10.3	84.4 ± 11.0	84.6 ± 9.2			
**Time spent standing (%)**	**O-RAGT**	10.5 ± 7.9	11.5 ± 8.3	10.3 ± 6.9	0.838	0.431	0.030
	**CON**	12.5 ± 7.6	12.2 ± 8.4	11.4 ± 6.6			
**Time spent stepping (%)**	**O-RAGT**	3.2 ± 3.0	5.2 ± 3.3	4.5 ± 3.1	3.673	0.032*	0.120
	**CON**	3.5 ± 2.9	3.3 ± 2.9	3.4 ± 2.7			
**Steps (n)**	**O-RAGT**	2754 ± 2809	4484 ± 3192	4105 ± 3350	2.720	0.090	0.092
	**CON**	3305 ± 3158	3231 ± 3159	3188 ± 3308			
**Number sit-to-stand transitions (n)**	**O-RAGT**	34 ± 11	40 ± 17	44 ± 14	1.825	0.173	0.063
	**CON**	39 ± 16	38 ± 17	36 ± 15			

Abbreviations: BL, baseline; CON, Control; O-RAGT, *Over-ground Robotic-Assisted Gait Training; PI, post-intervention; 3PI, three-month post-intervention*

*Significant Condition by Time interaction (p<0.05)
